# A Novel Technique for Inguinal Hernia Repair in Patients With Nonmalignant Ascites

**DOI:** 10.7759/cureus.75401

**Published:** 2024-12-09

**Authors:** Claire Dalby, Sinead L Brenner, Ahmed M Sharata, Nicolette M Winder, Paul C Kuo

**Affiliations:** 1 Surgery, Morsani College of Medicine, University of South Florida, Tampa, USA

**Keywords:** ascites, inguinal hernia, open inguinal hernia repair, postoperative outcomes, surgical technique

## Abstract

Introduction: We report a novel approach to open inguinal hernia repair in patients with known ascites in which the cord, hernia sac, and attached testicle on the affected side are repositioned into the retroperitoneum through the inguinal ring. By avoiding invasion of the peritoneum and limiting dissection of the sac off the spermatic cord, the risk of ascites leak and testicular ischemia is theoretically decreased.

Methodology: This is a retrospective case series report. Medical records of patients with ascites (*n* = 7) who underwent this novel repair technique were reviewed. All surgeries were performed at a tertiary referral center between January 2022 and January 2024.

Results: This surgical technique was performed on seven patients with inguinal hernias, all of whom had known ascites. Of these seven patients, none experienced postoperative inguinal hernia recurrence or ascitic leakage. However, three developed scrotal hematomas, and one experienced scrotal edema and postoperative constipation.

Conclusions: This technique may help reduce the risk of recurrence and ascitic leakage in this specific patient population.

## Introduction

The treatment modality and timing of inguinal hernia repair in patients with ascites are controversial, given the wide range of postoperative morbidity and mortality rates in this population [1]. Previously, surgical intervention was only considered when a complication such as strangulation or incarceration arose. More recently, research has shown that elective surgical repair is not only safe but also has favorable outcomes compared to emergent repair [2,3]. Even with these favorable outcomes, typical inguinal hernia repair in patients with ascites has the potential for complications such as infection and ascites leak, which may lead to significant associated morbidity and mortality [4]. Here, we present our experience of using a novel technique for non-emergent open inguinal hernia repair in patients with ascites.

We report a novel approach to open inguinal hernia repair in which the cord, hernia sac, and attached testicle on the affected side are repositioned into the retroperitoneum through the inguinal ring. The patients on which we performed this technique presented with scrotal inguinal hernias and had potentially prohibitive medical comorbidities including known ascites due to various etiologies. The rationale for performing this technique is it may reduce the risk of postoperative complications in this specific patient population. By avoiding invasion of the peritoneum and limiting dissection of the sac from the spermatic cord, the risk of ascitic leakage and testicular ischemia is theoretically reduced.

## Materials and methods

The medical records of patients (*n* = 7) undergoing this procedure between January 2022 and January 2024 were reviewed retrospectively. Patients were included if they (1) had ascites of nonmalignant etiology, (2) had at least one inguinal hernia that was surgically repaired by the novel technique described below, and (3) were over the age of 18. Patients were excluded if they did not have ascites, had ascites of a malignant etiology, did not have an inguinal hernia for which they underwent surgical repair, had undergone an inguinal hernia repair but were not performed using the novel technique described below, and were under the age of 18. All seven patients in this cohort had symptomatic hernias, with patients experiencing a variety of symptoms, including inguinal pain and/or discomfort, persistent scrotal swelling, and an enlarging inguinal hernia. Patients were given the option of expectant nonsurgical management for their inguinal hernias during the preoperative visit. Demographics, age, gender, body mass index (BMI), etiology of ascites, Child-Pugh score, Charlson Comorbidity Index, and hernia laterality were reviewed. Data were presented as mean +/- standard deviation. Preoperative, operative, and postoperative notes were reviewed. This study, identified as IRB ID STUDY007155, was exempted from IRB review by the University of South Florida's IRB/Research Integrity and Compliance Committee.

To repair the inguinal hernia in this novel technique, the patient was placed in the supine position. After adequate general anesthesia and antibiotics were administered, and a time-out was performed, a Foley catheter was placed. The lower abdomen, groin, penis, and scrotum were prepped and draped in a sterile fashion. A standard inguinal hernia incision was made at the affected lower quadrant(s). The subcutaneous tissue and Scarpa’s fascia were divided. The external oblique fascia was identified and incised. The external ring was then obliterated. Next, the flaps of the external oblique fascia were developed superiorly and inferiorly. The ilioinguinal nerve was identified and preserved. The spermatic cord was encircled at the level of the pubic tubercle and controlled with a Penrose drain, and then the cremasteric fibers were divided using electrocautery. Next, the hernia sac, filled with ascites, and spermatic cord were identified and dissected down to the scrotum and the testicle in a blunt fashion. Careful dissection and separation of the hernia sac were carried out to avoid inadvertent opening of the peritoneum. The cord, sac, and testicle were delivered into the operative field and repositioned into the retroperitoneum through the internal ring. The scrotum was then everted and explored for bleeding. Hemostasis was achieved using electrocautery and/or tissue sealant. A drain may be placed through the base of the affected scrotum. A standard Bassini repair of the inguinal floor was performed using interrupted nonabsorbable sutures, and the internal ring was closed with sutures. A standard 10 cm x 14 cm permanent mesh was then sutured into place, covering the repair area. Subcutaneous tissue, Scarpa’s fascia, and skin were then reapproximated using absorbable sutures. The patient was typically discharged home from the post-anesthesia care unit.

## Results

**Figure 1:**
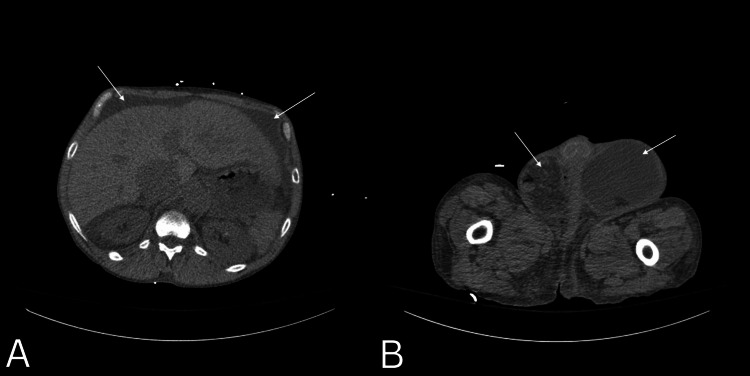
Preoperative axial computed tomography (CT) scan of a 56-year-old male patient, with ascites from heart failure. White arrows demonstrate ascites (A) and bilateral inguinal hernias (B).

**Figure 2:**
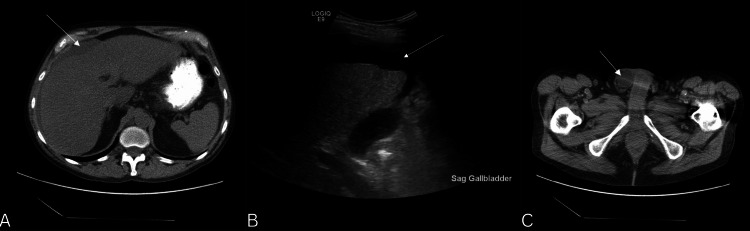
Preoperative axial CT scan (A and C) and preoperative sagittal right upper quadrant ultrasound (B) of a 62-year-old male, with ascites from alcoholic cirrhosis. White arrows demonstrate ascites (A and B) and a right inguinal hernia (C).

This technique was performed on seven patients with scrotal inguinal hernias, ranging in age from 56 to 82 years (Table 1). All patients were male with BMIs between 20 and 30. Evidence of preoperative inguinal hernia(s) and ascites was demonstrated on imaging (Figures 1-2) or physical examination. The etiology of ascites in these patients included hepatic cirrhosis, congestive heart failure on ventricular assist devices, and end-stage renal disease on peritoneal dialysis. Two of our patients underwent ascites drainage in the months before surgery. One was an 82-year-old male with alcoholic cirrhosis who had ascites drainage two and a half weeks before surgery, and the other was a 62-year-old male with alcoholic cirrhosis who had ascites drainage three and a half months before surgery. Several of the patients in this cohort had additional comorbidities, including diabetes mellitus, hypertension, hyperlipidemia, anemia, arrhythmias, and obstructive sleep apnea. Two patients in this cohort were former cigarette smokers. These seven patients had a Charlson Comorbidity Index ranging from 3 to 9, with one patient scoring 3 points, two patients scoring 4 points, one scoring 5 points, two scoring 7 points, and one scoring 9 points. Two patients were classified as Child-Pugh class A, four as class B, and one as class C. Of these seven patients, three had right inguinal hernias, two had left inguinal hernias, and two had bilateral inguinal hernias repaired by our novel technique.

**Table 1:** Demographics of patients who underwent our novel open inguinal hernia repair technique. SD, standard deviation; BMI, body mass index

Variable	Value
Age (years), mean (SD)	66.3 ± 9.8
BMI (kg/m^2^), mean (SD)	23.9 ± 3.1
Cause of ascites	
Heart failure	3
Alcoholic cirrhosis	2
End-stage renal disease on peritoneal dialysis	2
Child-Pugh classification	
A	2
B	4
C	1
Charlson Comorbidity Index	
3	1
4	2
5	1
6	0
7	2
8	0
9	1
Inguinal hernia site	
Right	3
Left	2
Bilateral	2
Length of clinic follow-up (months), mean (SD), range	1.3 ± 1.1, 0.5-3

**Figure 3:**
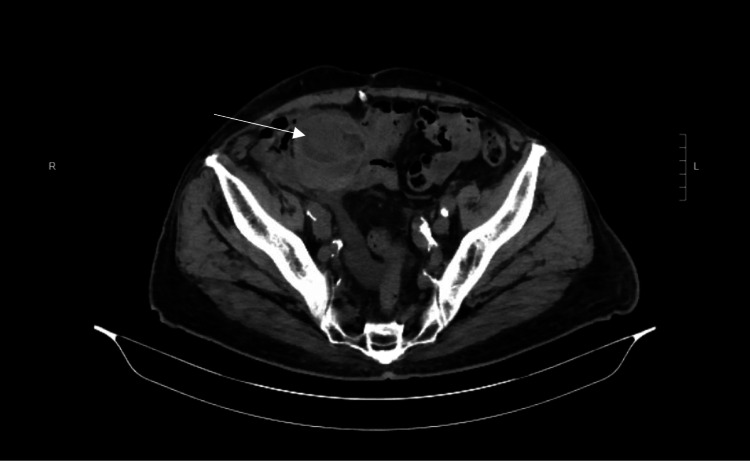
Postoperative axial CT scan of a 78-year-old male patient, with ascites from end-stage renal disease on peritoneal dialysis and a right-sided indirect inguinal hernia, following the described inguinal hernia repair. The arrow indicates the retroperitoneal testicle.

Postoperatively, there was no inguinal hernia recurrence in any patient in this cohort. No ascites leak or tear or the hernia sac occurred in this cohort. If the inadvertent opening of the hernia sac, filled with ascites, were to occur, we would need to address this intraoperatively by immediately closing the tear with an absorbable suture and attempting to evacuate any leaked ascites. Figure 3 demonstrates the retroperitoneal testicle on a computed tomography scan following hernia repair with this technique. Patients were followed via general surgery clinic visits for an average of five weeks postoperatively. Length of postoperative follow-up time ranged from two weeks to three months. Minor complications occurred in five patients in this cohort. Postoperative scrotal hematoma occurred in three of seven patients, with one patient also experiencing scrotal seroma, which was managed conservatively. One patient experienced an intraperitoneal hematoma after resuming anticoagulation postoperatively, with no concerns for ongoing acute bleeding at follow-up. One patient experienced minimal scrotal edema as well as postoperative constipation, leading to abdominal pain and a postoperative visit to an outside emergency department. This patient’s constipation was resolved with the implementation of a bowel regimen. Several patients in our cohort were readmitted to the hospital after their operation for chief complaints unrelated to their inguinal hernia repair. The first patient to undergo this operative technique was admitted to the hospital several times for complications of his congestive heart failure and eventually passed away nine months after his operation as a result of his heart failure. Another patient was admitted to an outside hospital with chest pain and urinary retention after a COVID-19 infection six months after his operation. A third patient was admitted for a thoracoabdominal aortic aneurysm with endoleak, a fourth for infection of the driveline of his left ventricular assist device, and a fifth for a motor vehicle crash. These frequent hospital admissions unrelated to our hernia repair suggest that while these are sick patients with significant comorbidities, this novel surgical technique for open inguinal hernia repair carried little surgical morbidity for our cohort.

## Discussion

Inguinal hernias in the patient population with ascites have historically been treated with nonsurgical management unless the patient was presenting in an emergent circumstance. However, in the last few years, this population has shifted towards support for elective surgical inguinal hernia repair [2]. A prior study examining the outcomes of open inguinal hernia repair with mesh in patients with ascites specifically caused by liver cirrhosis found that there was no 30-day postoperative mortality. However, two of the 22 patients experienced hematoma and one experienced scrotal swelling [3]. While this study had similar outcomes to our cohort, with some minor complications but no major complications or postoperative mortality, it was conducted only in patients with ascites from liver cirrhosis and ran the theoretical risk of causing an ascites leak via tearing of the hernia sac. One case report describes a patient with liver cirrhosis and ascites who successfully underwent bilateral inguinal hernia repair via a transabdominal preperitoneal approach, with closure by barbed sutures [5]. While this case report describes the successful use of the transabdominal preperitoneal approach, with no major complications, it also runs the theoretical risk of causing an ascites leak if peritoneal closure is incomplete. In another study in which laparoscopic totally extraperitoneal repair was attempted in 17 patients with inguinal hernia and ascites from liver cirrhosis, rupture of the peritoneum occurred in four cases, while no patients in our cohort experienced this complication [6].

The approach implemented in this patient cohort has proven to be a safe technique that minimizes manipulation of the hernia sac. By avoiding peritoneal invasion and limiting dissection of the hernia sac from the spermatic cord, the risk of ascites leakage and testicular ischemia is theoretically reduced. None of the patients included in this study developed postoperative ascites leak, infection, or testicular pain.

We have seen that elective inguinal hernia repair using this modified approach is safe and has minimal complications. Our study is limited by the small number of patients included in this cohort as well as the short duration of follow-up at this time. Replicating this study with a larger sample size would not only increase the power of the study but also allow us to further investigate outcomes based on different Child-Pugh classes and the benefit of optimizing ascites in the preoperative setting. There is minimal literature regarding the long-term effects of repositioning the testicle into the retroperitoneum in an adult male. Men with a history of an undescended testicle have been found to have a higher risk of developing testicular cancer and lower spermatogenic function than men whose testicles descended normally, which raises the question of whether repositioning the testicle into the abdomen in an adult male would have similar risks [7]. This study will therefore require long-term follow-up to determine potential complications of a retroperitoneal testicle, including the potential thermoregulatory and carcinogenic effects this may have. Ultimately, this technique could decrease morbidity and mortality compared to the current standard elective approach. 

## Conclusions

We present our experience utilizing a novel approach to open inguinal hernia repair in seven patients with nonmalignant ascites. In this approach, the spermatic cord, hernia sac, and attached testicle are manually repositioned into the retroperitoneum through the internal ring. There has been no hernia recurrence to date in this patient cohort. While some patients experienced minor complications, including scrotal hematoma, seroma, or edema, no patients experienced postoperative ascites leak or surgical site infection. Despite a cohort of patients with significant medical comorbidities who required several hospital admissions unrelated to the operation, this novel approach to hernia repair carried little surgical morbidity for our patients. Therefore, we conclude that this technique has the potential to improve outcomes in patients with ascites undergoing elective inguinal hernia repair.
